# The evolving trajectory of conjunction use in the ELT research articles

**DOI:** 10.3389/frma.2024.1337836

**Published:** 2024-11-28

**Authors:** Marzieh Safari, Fatemeh Mahdavirad

**Affiliations:** English Language and Literature Department, Faculty of Language and Literature, Yazd University, Yazd, Iran

**Keywords:** academic writing, cohesion, conjunction, research articles, old papers, modern papers

## Abstract

The present study was aimed at a diachronic investigation of conjunction as a grammatical cohesive device in ELT research articles. A total number of 100 research articles concentrating on teaching writing skills in the EFL context, and were released in two extremes of 1980–82 and 2020–22 were selected. The caution was taken to choose the papers which were written by expert English writers. Working within a descriptive-analytical framework, the type and frequency of conjunctions used in the articles were examined. The results depicted that the frequency of conjunctions increased at the expense of dropping in their variation. Having looked more closely, we found that particular conjunctions grew in use while the others fell out of the writers' favor. This pattern was also visible in the studies in which non-English writers' research articles were examined. This change, therefore, is attributed to the development of the speech community and the rise of non-English writers. Eventually, we concluded that it might be better to revise our material in ESP classes to adapt to the changes in a speech community.

## 1 Introduction

Discourse has been developed as a result of lengthy rhetorical practice and its conventions reflect the changing requirements of the audience (Hyland, 2004). Tracing discourse over a long time can shed light on the evolution and adaption of a language in a speech community. The use of cohesion for creating relations in discourse is also likely to change across history. The present research aims to study the changes in conjunction use, one of the subcategories of cohesion, within the 1980–2022 years across the research articles in English language teaching field.

Conjunctions or transitions in Hyland's words are metadiscourse markers that promote a sensation of semantic relations between sentences and highly appropriate use of them is a significant characteristic of academic writing (Hyland and Tse, 2004). Given their multifarious nature, conjunctions could enrich the facts, disagree with or refute the previous claims, set the scene for meeting a particular outcome, or, organize the narrated events in logical order. Such indispensable importance in bridging and strengthening the relations has always been subjected to tremendous research. An increasing body of research concentrated on comparison between the writings of L1 and L2 users of English, indicating some degrees of conjunction misuse among L2 writers of English (Field and Oi, 1992; Granger and Tyson, 1996; Tapper, 2005; Ishikawa, 2011; Lei, 2012; Gao, 2016; Chen, 2006).

In contrast, some researchers discarded the concept of non-English writers and placed value on academic expert writers apart from their L1. Their scrutiny dictates that academic writing needs to be practiced and is independent of the writers' mother language (Bocanegra-Valle, 2014; Zhao, 2017). Conjunctions were also compared within the disciplines and it has been concluded that in soft science, more connectors are used (Hyland and Tse, 2004; Peacock, 2010).

The contrastive studies in conjunction use are mainly concentrated on the difference between native and non-native writers or stress how different they are used in a range of genres. In this study, we shifted away from the regular traditions and adopted a new perspective. We sought the conjunctions' variation over time; to our best knowledge, it has not been investigated so far. The logic behind this exploration is, on one hand, the studies which showed that non-English native writers are not necessarily disadvantaged. Zhao (2017) in particular, did not find the discrepancy in conjunction use between L1 and L2 scholars as they knew the conventions. She put the misuse of conjunctions down to the unfamiliarity with academic language, not the writers' L1. On the other hand, we know that discourse develops to match its users' needs. Given these facts, analysis of conjunctions as expert writers use them can lead us to realize how conjunctions are contextualized and modified to live up to their expectations. We need to bear in mind that apart from the creation of integrity and continuity in a text, conjunctions are like signs that show how ideas are organized. The odds are that pursuing these signs reveals the mindsets and the way of arguing have been developed in long-lasting practice among the speech community members. The findings would contribute to a list of the most used conjunctions in articles and a practical guide in ESP material development. This guide profits the novice writers who are striving to find their feet in an increased competitiveness of research positions. Thus, the present study addresses the following research question:

What are the types and frequency of conjunctions used in modern vs. old English research articles?

In the following, first, some studies with relevant content are reviewed and then the obtained results are presented through diagrams and their interpretation. Next, the results are subjected to discussion and finally, the conclusion and implication of the study are outlined.

## 2 Review of the related literature

### 2.1 Conjunction as a subcategory of cohesion

Cohesion is generally defined as continuity between different parts of a certain text which could be manifested by cohesion devices (Halliday and Hasan, 1976: 299). These devices are categorized as lexical and grammatical each of which has subcategories. In terms of lexical devices, they are labeled as reiteration (repetition, synonymies) and collocation (co-occurrence of lexical items). The subcategories of grammatical cohesion are reference, substitution, ellipsis, and conjunction (Halliday and Hasan, 1976). Having emphasized that there is no definite classification, Halliday and Hasan (1976) came up with a comprehensive classification system; additives, adversatives, casuals, and the temporal (Halliday and Hasan, 1976, p. 245).

Considering the coordinated conjunction as single units and discarding their role in maintaining cohesion, Halliday and Hasan (1976) introduced additive as conjunctions only with an addition nature. These conjunctions link a sentence or paragraph to the previous sentence when attaching new information seems necessary. The adversatives induce a contrast from what has been acknowledged, signaling a controversy. The casuals or the resultative bring up conditions under which an outcome happens or pave the way for coming to conclusions. Ultimately, the temporal indicates the sequence between the findings.

This classification system has inspired other researchers. Quirk et al. (1985) introduced a seven-subcategory system. In his grouping, the temporal expanded to listing, summative, and transition. Casuals also turned into inferential and resultative; however afterward, Biber et al. (1999) discarded their duality and merged them again. He discriminated opposition from contrast/concession in adversatives and presented them as independent categories.

Halliday and Hasan's (1976) taxonomy benefits the study by giving an elaborated taxonomy of conjunctions based on their function in context. This taxonomy allows us to compare the changes in conjunction with use over time. Secondly, we needed to draw on a classification commonly used by other studies to interpret our findings. Although our efforts to find studies focused on chronological changes of conjunctions failed, we found similar studies in which Halliday and Hasan's (1976) framework was used prominently.

### 2.2 Conjunctions across L1 and L2 writers of English

Numerous types of research have been devoted to weight non-native-English writers' essays against English- native writers' ones. These studies show close similarities; however, it seems that some studies outperformed as they assured that the writers have the same educational level whether they are L1 or L2 users of English. Field and Oi (1992) and Hinkel (2003), for instance, carried out their study among students with English-native and non-English-native backgrounds. It seems that these studies take the academic experience as an indicating factor into account.

Field and Oi (1992) compared conjunctions in essays written by Cantonese and Australian students. Having concluded the overuse of these devices in Cantonese essays, they pointed out the importance of presenting conjunction with their real syntactic and semantic features. Hinkel (2003) exclusively explored the concessive adverbs in texts written by students in five universities with five different background languages and Americans were one of those groups. The results proved the significant use of conceiving adverbs in Americans' essays. Afzaal et al. (2019) analyzed the cohesion devices in Pakistani English newspapers. Their findings showed that additives are highly frequent in the texts. Causals took the second place, and adversatives followed them. Safari and Mahdavirad (2021) examined conjunctions in 100 abstracts written by English native researchers and non- English native researchers (Iranians) who were academically experienced in writing. The analysis revealed that additives frequented tremendously by Iranian researchers while the adversative use was half as many as the adversatives by English native researchers. They concluded that Iranians are influenced by the rhetorical patterns embedded in their L1 writing culture. Afzaal et al. (2021) compared 20 introductions in the master's thesis of Chinese and American graduates in the use of metadiscoursal elements proposed by Hyland (2005). His investigation showed that the Chinese students comparably use fewer number of these elements. One reason that he put forward was that in Chinese culture the reader is kept more accountable and the responsibility of reading is on the reader. Alawerdy and Alalwi (2022) carried out a conventional pre-test/post-test design in which the treatment was explicit education of cohesion devices in paragraph development. The results confirmed the significance of instruction in correct use of conjunctions in EFL students' essays. Saeed (2023) analyzed conjunction use in the academic writing of EFL students. The finding confirmed that students used the additives excessively.

In the following studies, the non-English native writers' drafts have been compared with papers published in international journals or essays developed by English native writers but not necessarily with the same educational level. Milton and Tsang (1993) studied conjunction in assignments written by university students in Hong Kong and compared them with English published essays. The study illustrated that students use conjunctions excessively while the context is cohesive. Granger and Tyson (1996) concluded overuse of adversatives when they investigated the argumentative writings written by French advanced learners and English natives. As a result, they recommended taking into account the semantic properties, and syntactic positions and distinguishing the styles. Lei (2012) found adversatives more problematic than additives in Chines Ph.D. students' dissertations when she contrasted them to articles published in international journals. Adversative use was restricted to a low number of them. Chen (2006) explored conjunctions across MA TESOL students' papers and published papers in prestige international TESOL journals. The results perfectly showed the inappropriate use of conjunctions by Taiwanese students.

Meanwhile, Zhao (2017) came up with the results of a new study, stressing the retooling of examination. Rather than comparing L2 novice writers' drafts with prestigious international journals, He believed researchers should draw the spotlight on L1 and L2 writers who participate in courses on academic paper writing. Having analyzed conjunction realization among L1 and L2 novice writers and L1 and L2 expert writers, Zhao (2017) concluded that the first issue of novice writers whether with L1 or L2 backgrounds is rooted in their insufficient knowledge of academic conventions. These results were in fulfillment of studies that dismissed the disadvantage of English language as a lingua franca among academicians (Ferguson et al., 2011).

Hyland and Tse (2004) found higher use of transitions in soft science as in this science the proofs and quantitative data are not as concrete as it is in hard science. Liu (2008) depicted that the frequency of linking adverbials used in academic writing and spoken discourse is higher than its frequency in fiction and news. Peacock (2010) concluded that the higher frequency of linking adverbials in science papers than in non-science ones reveals the variation of conjunctions across disciplines.

## 3 Materials and methods

Given the background study that has just been reviewed, our corpus needs to be consistent in terms of genre, discipline, and the writers' competency. As for why, we concentrated our study on a collection of 100 articles devoted fully to teaching writing skills in EFL and released in two extremes of 1980-82 and 2020-22 equally. In the process of data collection, first, the journals in academic writing were chosen, and precaution was taken to be chosen from journals which were indexed in well-known databases. Then the search was narrowed down to the papers that exclusively targeted teaching academic writing. After this filter, we needed to ensure that the writers were academically experienced because we intended to analyze the true and accepted way of using conjunctions apart from the nationality of the writers. To serve this purpose, we looked up the writers' biographies on Google Scholar to gain an understanding of their reputations. At this stage, we chose the papers whose writers had an outstanding number of papers. it is worth noting the papers with more than three writers were dismissed and among multiple writer papers, only the corresponding writer was considered. All these papers are precisely addressed in the Appendix.

Halliday and Hasan's (1976) classification system seems straightforward, we needed a more detailed framework to put it into practice. In result of our search, the taxonomy of Liu (2008) has proven to be fruitful. Liu (2008) consulted eight grammar books and provided a 110-linking adverbials list. This list was examined by exploring their variation and frequency in five registers: spoken, academic writing, news, fiction, and others. What has been given to birth was a comprehensive list flexible in every context and register. This taxonomy was also employed by Lei (2012); Wang (2014); Gao (2016) and Safari and Mahdavirad (2021). Table 1 shows the Liu's (2008) taxonomy.

**Table 1:** Liu's (2008) taxonomy.

**Type**	**Subcategory**	**Example**
Additive	Emphatic	Also
	Appositional/reformulation	Namely
	Similarity comparative	Likewise
Adversative	Proper adversative/concessive	However
	Contrastive	Actually
	Dismissal	Anyway
Causal/resultative	General causal	So
	Conditional causal	Otherwise
Sequential	Enumerative/listing	First
	Simultaneous	Meanwhile
	Summative	To sum up
	Transitional to another topic	By the way

After adopting the well-organized taxonomy of Liu (2008), a preliminary examination of every conjunction was conducted to check whether all of them operate as sentence connectors. This examination was performed by considering the relevant and surrounding sentences. Then, the frequency of every item was entered into Excel 2020 to calculate the sum of items in each sub-corpus of Liu's taxonomy. In this process of counting, we came across conjunctions whose incidence was comparably low and they were not worth being considered in the study. As a result, they were removed from the rest of the study.

## 4 Results

Liu's (2008) taxonomy includes110 connectors and these connectors were explored in the corpus study. Among these connectors, some of them have proven to be more functional in modern or ancient data. The reliability of the study was assured by intra-rater and inter-rater reliability after a time lapse of 1 week. To simplify analyzing and interpretation of the results, the highly used connectors were chosen and studied. In the following part, first, the identified conjunctions are compared within each series and then these conjunctions are compared between the series.

**Figure 1:**
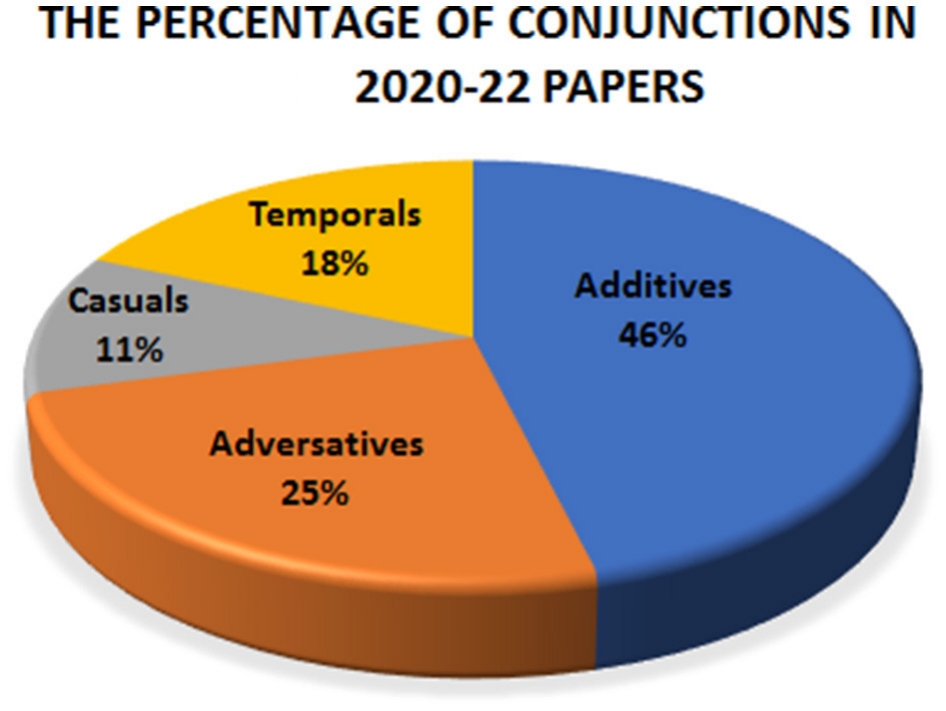
The proportion of conjunctions in 2020–22 papers.

The analysis of the data indicates that in 2020–22 papers, the additives constitute hardly one-half of the conjunctions, at 46%. The proportion of adversatives is almost half of the additives. On the other hand, casuals and the temporal make up for 18%, and 11% of the conjunctions respectively. Figure 1 compares the conjunctions in 2020–22 papers (Color should be used in every figure of this study in print).

**Figure 2:**
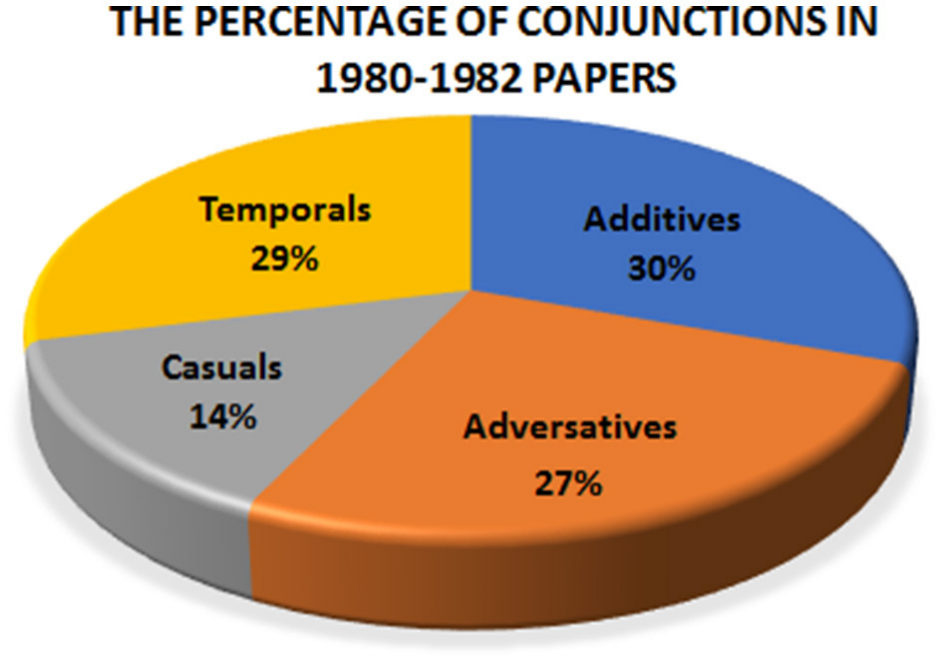
The proportion of conjunctions in 1980–82 papers.

**Figure 3:**
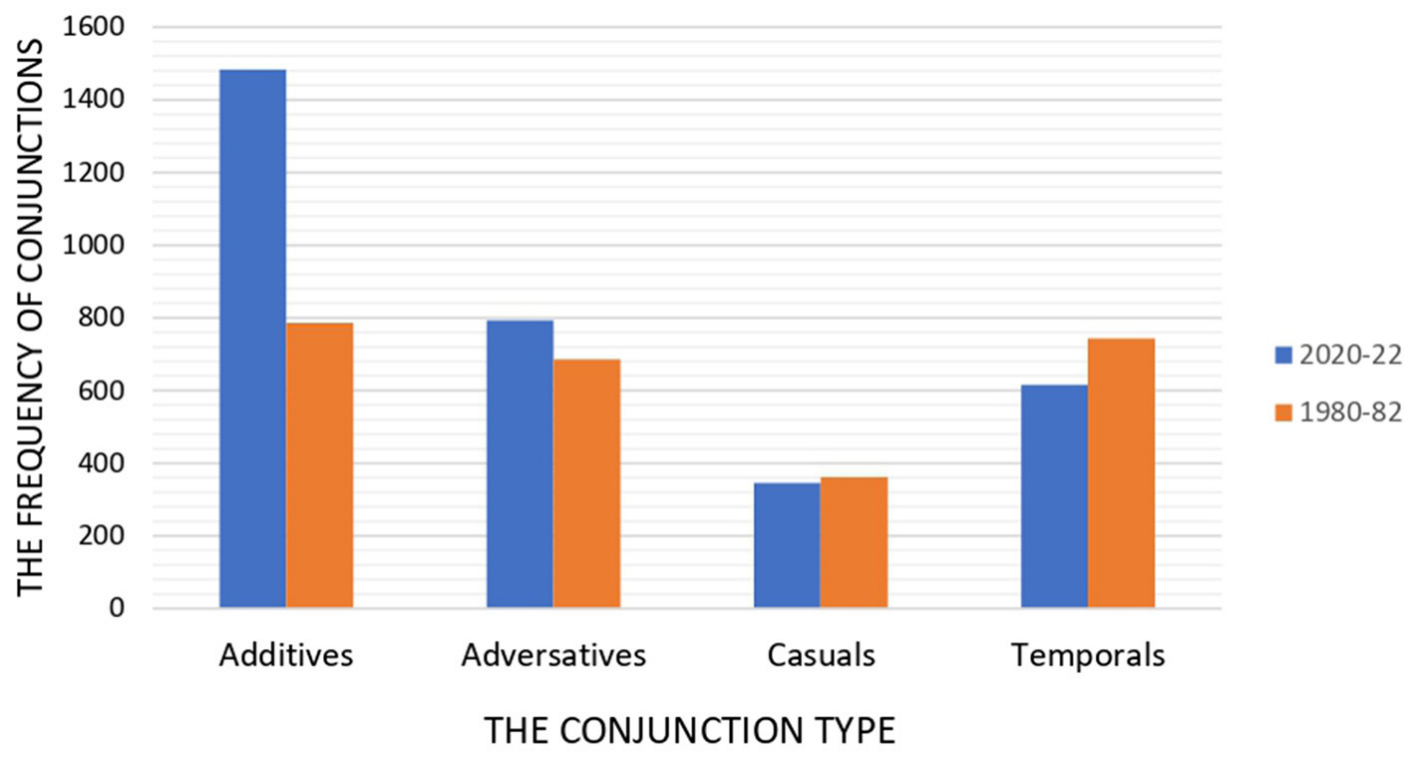
Frequency of conjunctions across 1980–82 and 2000–22 papers.

In contrast to the 2020–22 papers, it seems that there is some kind of balance in conjunction distribution in the 1980–82 papers. The percentage of additives, adversatives, and the temporal fluctuates between 27% and 30%, however, casuals are used much fewer, at 14%. Figure 2 compares the conjunctions in 1980–82 papers. Figure 3 compares the distribution of conjunctions in 2000–2022 and 1980–82.

In addition, the statistical analysis results showed that the conjunction use grew noticeably in recent papers. In old papers, 2,576 conjunctions were used whereas in modern articles, it reached to 3,211. A more detailed exploration of the data revealed that the incidence of additives and adversatives increased while the casuals saw a modest decline.

**Figure 4:**
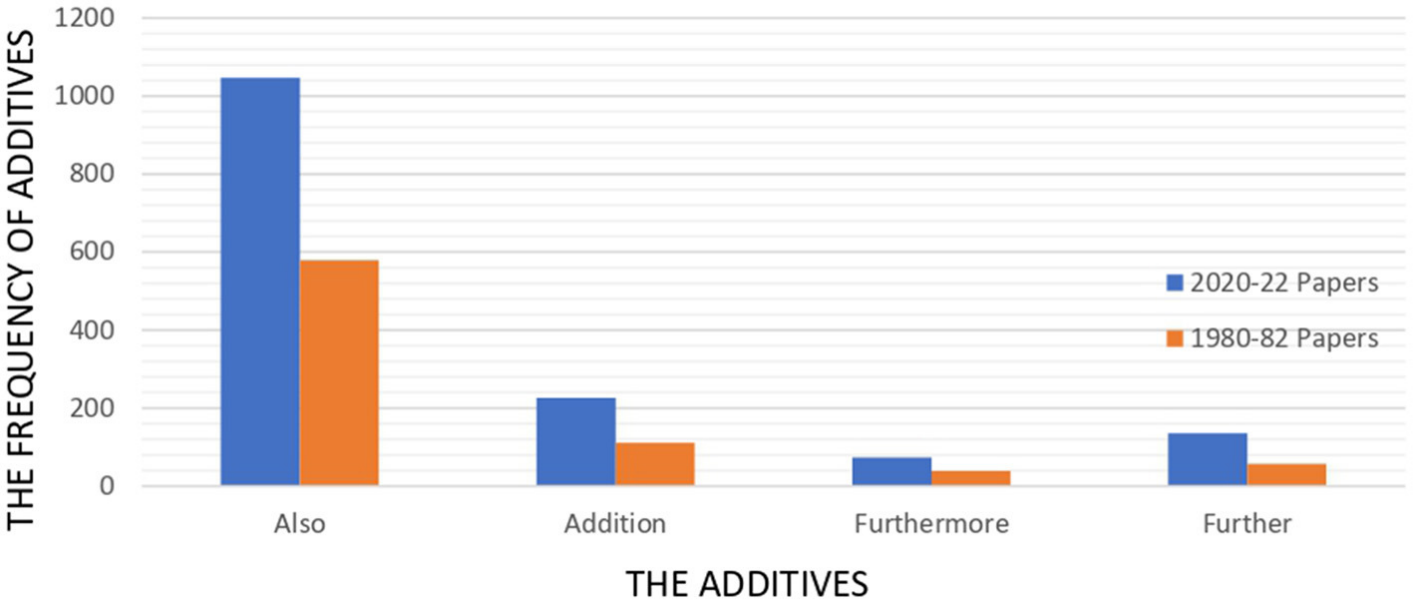
Frequency of additives across 1980–82 and 2020–22 papers.

Having looked at the details, it is realized that the frequency of “also” (“and also” and “not only … but also”) is considerable in 2020–2022 papers and 1980–1982, reaching to 1,047 and 579, respectively. “In addition” and “additionally” are in the second position, showing a huge gap with “also”. The use of “further” and “furthermore” were trivial, making up < 15% in both series. Figure 4 illustrates the distribution of additives within 2020–22 and 1980–82 papers.

**Figure 5:**
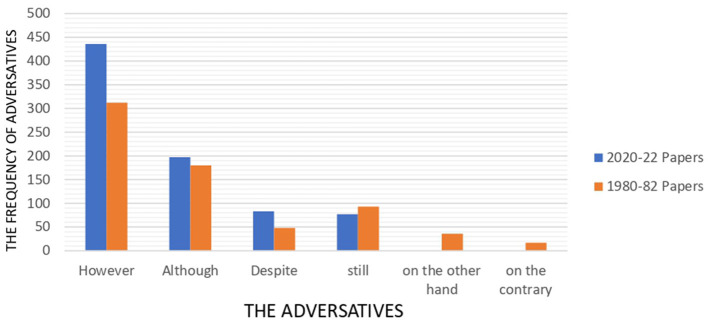
Frequency of adversatives across 1980–82 and 2020–22 papers.

In comparison to additives, adversatives rose slightly although the old papers show more variation of adversatives. In 2020–2022 papers, the adversative use is skewed heavily toward “however” and “although”, making up for about 80 percent of adversatives. In contrast, 1980–82 papers witnessed more different kinds of adversatives. “on the other hand” and “on the contrary, for instance, frequented in older papers while they were absent in the modern ones. Figure 5 illustrates the distribution of adversatives within 2020–22 and 1980–82 papers.

“However” stands out noticeably in both papers, reaching slightly blew 450 in 2020–22 papers and above 300 in 1980–82. “Although” was used quite equally in both series, around 175, and “Despite” was used 25 more in modern articles. The incidence of other adversatives was scarce in 2020–22 papers. The use of “still” fell from 93 to 77 and the next two adversatives did not appear at all while they were used 53 times in ancient papers.

**Figure 6:**
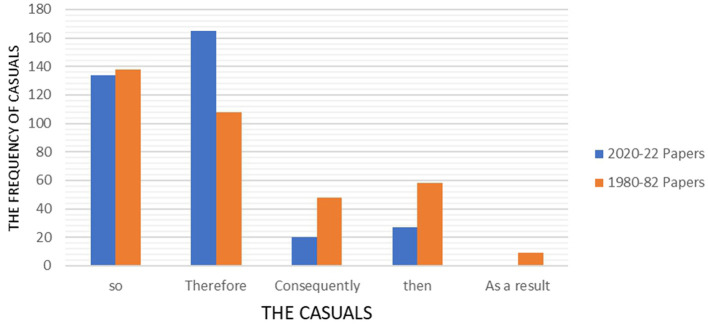
Frequency of casuals across 1980–82 and 2020–22 papers.

Despite the increasing pattern of additives and adversatives identified in modern papers, the casuals and temporal use fell modestly. The casuals in 2000–22 papers not only did drop but it also diminished in variation. Figure 6 illustrates the distribution of casuals within 2020–22 and 1980–82 papers.

**Figure 7:**
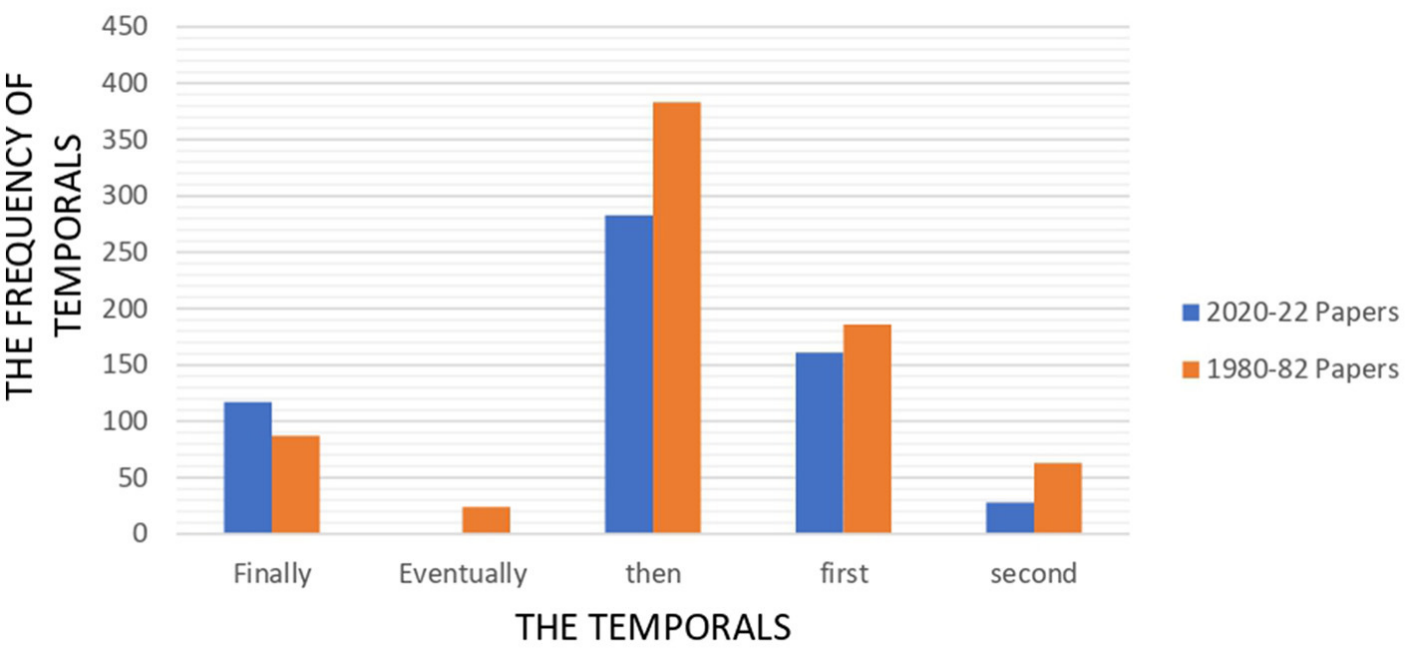
Frequency of the temporal across 1980–82 and 2020–22 papers.

The only conjunction in casuals which rose significantly in 2000–22 articles is “therefore” which soured from around 100 in 1980–82 to above 160. “so”, (“so”, “and so”, “so that”, “so as”) fluctuates around 135 in both series while the other casuals dropped drastically or reached to zero. “Consequently” and “then” cut by half and “as a result” were not found in new papers. The fall in the temporal is more significant, declining from 801 to 616. Figure 7 illustrates the distribution of the temporal within 2020–22 and 1980–82 papers.

“Then” is highly used in both series to order the events. The number of this connector fell from 400 to < 300 in the 2020–22 papers. “First”, “first of all, and “firstly” occupy the second position in both series, reducing from 161 to 186. “Finally” fell slightly and “Second” halved in new articles.

## 5 Discussion

The present investigation at the first stage illuminated that the 1980–92 papers have an almost equal proportion of conjunctions, whereas the additives took the lead in the 2020–22 papers, followed by the adversatives. The comparison of identified conjunctions in two series of data confirms the increase of conjunctions in recent years. This rise is owed to additives, in particular, and adversatives. To interpret what happened, we stated two assumptions, strong and weak, and then supplied them:

-The strong assumption is that the wider the speech community has grown, the more it has been influenced by the other nation's rhetorical practice.

-The weak assumption is that the longer the period between the released articles, the more some findings can help to elaborate the new findings.

For the strong assumption, we need to take a quick look at the articles which concentrated on the conjunction used by non-English-native speakers. Granger and Tyson (1996), reported that French natives tend to overuse additives and underuse adversatives. Moreover, Tapper (2005) found out that advanced EFL learners have a great tendency to overuse all conjunction types, particularly additives. In the study by Ishikawa (2011) also, it was shown that Asian research paper writers are more likely to overuse additives in their writings. In another study, Shirazi and Mousavi (2017), reported that the frequency of adversatives employed in papers written by Iranian academicians is half of the adversatives in English native writers' papers. In addition, Safari and Mahdavirad (2021) examined conjunctions in 100 abstracts. These studies which mainly targeted advanced, experienced non-English-native writers address a fact in their rhetorical practice. They excessively use additives to introduce new information, enrich the facts and as a result get their meaning across more persuasively and enticingly. Meanwhile, the study of adversatives used by non-English-native writers could get us another hint.

In the study by Kuzborska and Soden (2018) Chinese EFL learners' writings on three levels was compared. It was revealed that 'although', and 'however' are frequently used in high score texts. The results of the study by Aull and Lancaster (2014) indicated that 'however' and 'although' are frequently used in their three corpora under investigation: first-year learners' essays, upper-level learners' texts, and academicians' texts. It was also shown that first-year learners are more likely to use adversatives such as 'however' and 'though' which are more flexible while avoiding employing adversatives like 'nevertheless', 'in contrast, or 'on the other hand' that make a strong concession. Hinkel (2003), reported that concessive markers like 'although', 'even though, 'while', and 'whereas' were rarely employed in the writing of first-year natives and academically-advanced non-natives. She explained that “concession clauses are syntactically and semantically advanced subordinate constructions”, and are rarely found in the texts developed by both natives and non-natives. Safari and Mahdavirad (2021) found that not only the frequency of adversative halved in Iranians' abstracts in comparison to their counterparts, but also did it decline to some particular ones including “however” and “although”. And finally, what we found was a significantly increasing willingness to use “however” and “although” in the recent articles while the incidence of other adversatives was on the verge of being disappeared.

The more use of additives and adherence to the use of particularly limited adversatives are coming to be seen as a familiar pattern among non-English-natives. In addition, Hyland believes that the conventions of writing are determined by the academic community (Hyland, 2004). It seems that with the larger scale community of academic writing and the fast-growing number of papers that come out by L2 writers of English, the writing style is shifting away from the costumery style of conjunction use to a modern style, signaling more the style of non-English speakers. This ever-changing rhetoric practice is affected by the members and correspondingly influences the norms and expectations of the rhetoric.

The changes in casual use are quite chaotic. While “so” was still rife in 2000-22 papers, other casuals underwent remarkable changes. “Therefore” frequented massively in 2020-22 papers at the expense of “consequently”, “then”, and “as a result. This fall in variation is also tractable in the temporal conjunctions. Apart from “finally” which is frequented more in recent papers, the other variations reduced in number. The chances are that the shrinking of conjunction group is gaining ground as they ease the burden of their use in practice. In other words, the less the number of conjunctions, the easier they are to use.

The weak assumption cast some light on the lengthy interval of these two series of papers. Based on the practicality principle, no research can be independent and science has been built up as a result of numerous studies (Farhady, 2009). The released papers are at the two extremes of 1980–82 and 2020–22 with a 40 years interval. The recent papers need to recite more on the previous papers to increase standing in the eyes of the science community. Indeed, they attach more findings to confirm or refute their studies. The attachment of this information consistent with or in contrast to some findings or expectorations requires the employment of more connectors, namely, additives and adversatives. By a way of example, you can take a look at these extracts which has been drawn from an article:

*However, in addition to exploring the effectiveness of peer feedback in L2 writing development, the existing research on peer feedback has mainly focused on students' perceptions of and attitudes toward peer feedback*.

*Additionally, a case study of master's students' engagement with peer feedback in the academic writing context revealed that …*

*Additionally, consistent with the findings of research on teacher feedback, students deployed a variety of meta-cognitive and cognitive strategies …*

*However, this expectation may not always be fulfilled due to …*

*However, only focusing on the behavioral dimension is not enough to …However, previous research has revealed little about …*

Take also these extracts into consideration from another research article:

*Furthermore, little is known as to what types of multimodal writing should be prioritized in academic settings*.

*This observation may indicate that multimodal task performance involves more planning for language formulation and production than we have expected. Furthermore, academic multimodal texts*

*Furthermore, given recent studies finding facilitative effects of multimodal tasks on language proficiency and literacies, more research is necessary to*

*However, using multimodal tasks in second language (L2) classes often presents challenges because of varied definitions*

*However, studies taking the weak version of multimodality have offered …*

*However, we identified two different functions of multimodal tasks*.

*In terms of the linguistic mode, spoken words tends to be a more dominant method in meaning making than written words; however, it should be noted that …*

*We found that for multimodal writing, however, individual tasks were far more common …*

There is no denying that body of knowledge has developed considerably within 40 years and it has far-reaching effects on new studies. The necessity of presenting a well-documented and neat content make the writers resort to different from of additives and adversatives. If a study doesn't put up on a pre-planned scaffolding of other researches, it cannot come into being.

## 6 Conclusion

The present study evidence the claim that academic writing has a dynamic nature. We drew the spotlight on conjunctions used in 100 papers published in 2020–22 and 1980–82 and discovered that the incidence of additives and adversatives rose in recent papers whereas the casuals and the temporal fell slightly. Despite this contradiction in incidence, the reduction in variation is common in each group. In other words, whether the token of conjunction has increased or decreased in these four groups, their type is in steady decline. To find an answer for this change, the definition of academic writing as suggested by Hyland (2004) could be helpful: “academic writing is a policy of language that is established by the members of a scientific community” (Hyland, 2004, p. 12). Along with the increasing growth of non-English-native writers and the widening of the speech community, conjunction use is ushered in a concise and useful way. Seemingly, contraction of variation and adherence to particular connectors is a strategy that helps writers to cut through the burden of their use in academic papers.

The influence of L2 writers of English on the norms of connector use is particularly visible in additives and adversatives. Having dug the studies which compared the conjunction use within English and not-English writers, we found similar patterns in the advanced non-English-native writers' peppers. So-called “overuse of some additives and the high frequency of some particular adversatives in these studies were consistent with our results. To put it in nutshell, it is likely that the evaluation of non-native-English writers” papers with the benchmark of native-English writers' rhetorical practice doesn't lead us anywhere and the image of the natives' production as the standard practice is going to shatter.

Another driver of these results lies in the fact that the 2000022 papers were published 40 years later after the 1980–82 papers. The wider the background of studies in result of lengthy practice, the more the authors need to contribute and make reference to other studies. This is called intertextuality which has crucial importance in academic writing. The papers in 2000–22 take advantage of quite developed content, strategies, and beliefs, as to why more use of connectors is required to make contributions and citing to them.

Academic writing is a manner of communicative practice, and its conventions have been nurtured by its members. The scholars are well familiar with the conventions of writing in their discipline and know how to modify their practice to live up to the expectations of a speech community. This relation, however, is mutual and the scholars themselves affect the conventions. The different patterns of conjunction used in modern papers tracked down in the papers of non-native English writers were an example of these changes. With the development of the speech community, these cohesive devices mutated into a new pattern in practice.

This study has implications for syllabus design and material development. In the collection of data, we came to a shorter list of conjunctions. It is assumed, therefore, that this curtailed list could lighten the burden of their use and could come into effect in the short-term course of academic writing. Picking up the highly frequent conjunctions and their correct functions in the text would be more rewarding in writing papers rather than a long list. This list could also be considered as a benchmark for editors and reviewers to judge the researchers' papers. As it is explained, it seems inappropriate to set high standard of excellency as the norms of nonnative writing is dominating.

This study came to terms with some limitations and could not be flawless. First, it was boiled down to papers in “teaching English writing” to control the variable of discipline and cut the complexity of the study, however, inevitably it fell short of being accountable for each type of context. Secondly, the analysis was only carried out in written genre and the oral form was neglected. Considering lectures or conference presentations could enrich this study. On the other hand, some variables were not controlled and assumed to be neutralized in the process of data collecting like L1 background or natality of writers. As it is mentioned in some studies, the English language does not necessarily disadvantage non-English-native writers as academic writing is assumed to be a practice among the speech community members and depends on the competency of writers in academic writing (#3:5). However, for the further study, it is advisable to consider how L1 background of writers could influence the conventions of conjunction use.

## Data Availability

The original contributions presented in the study are included in the article/Supplementary material, further inquiries can be directed to the corresponding author.
