# Supplementary material for: The evolving trajectory of conjunction use in the ELT research articles
Source: Front Res Metr Anal. 2024 Nov 28;9:1337836. doi: 10.3389/frma.2024.1337836 (PMC11634842; doi:10.3389/frma.2024.1337836)
Supplement: Supplementary file 1 [file Data_Sheet_1.docx]

**Appendix A (1980-1982 Corpus)**

Beard, R. E. (1981). On the question of lexical regularity. *Journal of Linguistics*, *17*(1), 31–37.

Bruce Cassie, J. R., & Palmer, D. (1981). Quantity and nature of continuous prose writing in English classes—grades 9–13. *Interchange, 12*(4), 64-77.‏

Caldwell, D. (1982). The graduate student as a creative writer: Teaching and learning with a drama project. *Die Unterrichtspraxis / Teaching German, 15*(1), 87–94.

Cardelle, M., & Corno, L. (1981). Effects on second language learning of variations in written feedback on homework assignments. *TESOL Quarterly, 15*(3), 251-261.‏

Carpenter, C., & Hunter, J. (1981). Functional exercises: Improving overall coherence in ESL writing. *TESOL Quarterly*, *15*(4), 425–434.

Carpenter, C., & Hunter, J. (1981). Functional exercises: Improving overall coherence in ESL writing. *TESOL Quarterly, 15*(4), 425-434.‏

Carrell, P. L. (1982). Cohesion is not coherence. *TESOL Quarterly*, *16*(4), 479–488.

Collins, J. L., & Williamson, M. M. (1981). Spoken language and semantic abbreviation in writing. *Research in the Teaching of English*, *15*(1), 23–35.

Cooper, T., & Morain, G. (1980). A study of sentence-combining techniques for developing written and oral fluency in French. *The French Review, 53*(3), 411-423.‏

Culpepper, M. M., & Ramsdell, R. (1982). A comparison of a multiple choice and an essay test of writing skills. *Research in the Teaching of English*, *16*(3), 295-297.‏

Daiute, C. A. (1981). Psycholinguistic foundations of the writing process. *Research in the Teaching of English*, *15*(1), 5-22.‏

Dobrin, D. N. (1982). What’s difficult about teaching technical Writing. *College English*, 44(2), 135–140.

Doyle, A. E. (1982). The limitations of cohesion. *Research in the Teaching of English, 16*(4), 390–393.

Dubin, F., & Olshtain, E. (1980). The Interface of Writing and Reading. *TESOL Quarterly, 14*(3), 353–363.

Duncan, J., Dye, C., Lazarus, J., Schwartzmann, D., Warner, J. A., & Hendin, R. (1981). Young adult literature: New writes of Passage. *The English Journal*, *70*(4), 76–79.

Edelsky, C. (1982). Writing in a bilingual program: The relation of L1 and L2 texts. *TESOL quarterly, 16*(2), 211-228.‏

Freedman, A. (1980). During not after: an untraditional approach to the teaching of writing. *English in Education, 14*(1), 2-9.‏

Freedman, A., & Pringle, I. (1984). Why students can’t write arguments. *English in education*, *18*(2), 73-84.‏

Freedman, C. (1981). Writing, Ideology, and Politics: Orwell’s “Politics and the English Language” and English Composition. *College English, 43*(4), 327–340.

Fulwiler, T. (1980). Journals across the disciplines. *The English Journal*, *69*(9), 14–19.

Gebhardt, R. (1980). Teamwork and feedback: Broadening the base of collaborative writing. College English, 42(1), 69-74.‏

Gebhardt, R. C. (1982). Initial plans and spontaneous composition: Toward a comprehensive theory of the writing Process. *College English*, *44*(6), 620–627.

Goldschmidt, M. (2014). Teaching writing in the disciplines: student perspectives on learning genre. *Teaching & Learning Inquiry: The ISSOTL Journal, 2*(2), 25–40.

Goodin, G., & Perkins, K. (1982). Discourse analysis and the art of Coherence. *College English, 44*(1), 57–63.

Hendrickson, J. M. (1980). The treatment of error in written work. *The Modern Language Journal, 64*(2), 216-221.‏

Herrington, Anne J. (1981). Writing to Learn: Writing across the disciplines. *College English,* *43*(4), 379–87.

Hill, S. S., Soppelsa, B. F., & West, G. K. (1982). Teaching ESL students to read and write experimental‐research papers. *TESOL quarterly, 16*(3), 333-347.‏

Holloway, D. W. (1981). Semantic grammars: How they can help us teach writing. *College Composition and Communication, 32*(2), 205-218.‏

Hubbard, S. A. (1982). Creative writing and ‘A’level English. English in Education, 16(1), 8-16.‏

Johns, A. M. (1980). Cohesion in written business discourse: Some contrasts. *The ESP Journal*, *1*(1), 35-43.‏

Johns, A. M. (1980). Cohesion in written business discourse: Some contrasts. *The ESP Journal, 1*(1), 35-43.‏

Johnson, S. T. (1981). The Ant and the grasshopper: Some reflections on prewriting. *College English, 43*(3), 232–241.

Knoblauch, C. H., & Brannon, L. (1981). Teacher commentary on student writing: The state of the art. *Freshman English News*, *10*(2), 1–4.

Larson, R. L. (1982). The “Research Paper” in the Writing Course: A Non-Form of Writing. *College English*, *44*(8), 811–816.

Maimon, E. P. (1982). Writing across the curriculum: Past, present, and future. *New Directions for Teaching and Learning*, 12, 67-73.‏

Matthies, B. (1980). The Non-Native Speaker of English Learns to Write—Somehow. *Canadian Modern Language Review*, *36*(4), 713-723.‏

McCabe, P. P. (1981). Cohesive ties in text. *Language Arts, 58*(8), 945–946.

McKee, E. (1981). Teaching writing in the second language composition/conversation class at the college level. *Foreign Language Annals, 14*(4), 273-278.‏

McKee, E. (1981). Teaching writing in the second language composition/conversation class at the college level. *Foreign Language Annals*, *14*(4), 273-278.‏

McTeague, F. (1980). An Investigation of Secondary Student Writing Across the Curriculum and Some Suggestions for School. *Language Policies,6*, 184-192.‏

Menyuk, P., & Flood, J. (1981). Linguistic competence, reading, writing problems and remediation. *Bulletin of the Orton Society*, *31*, 13-28.‏

Moran, C. (1981). Teaching Writing/Teaching Literature. *College Composition and Communication*, *32*(1), 21–29.

Nattinger, J. R. (1980). A Lexical Phrase Grammar for ESL. *TESOL Quarterly, 14*(3), 337–344.

Odell, L., & Goswami, D. (1982). Writing in a non-academic setting. *Research in the Teaching of English,16*(3), 201-223.‏

Selzer, J. (1980). Another look at paragraphs in technical writing*. Journal of Technical Writing and Communication, 10*(4), 293-301.‏

Sommers, N. (1982). Responding to student writing. *College composition and communication, 33*(2), 148-156.‏

Watson, C. B. (1982). The use and abuse of models in the ESL writing class. *TESOL quarterly, 16*(1), 5-14.‏

Witte, S. P., & Faigley, L. (1981). Coherence, Cohesion, and Writing Quality. *College Composition and Communication*, *32*(2), 189–204.

Zamel, V. (1981). Sentence combination: Writing and combining standard English sentences: Book II.‏ *TESOL Quarterly, 15*(4), 468–471.

Zamel, V. (1982). Writing: The process of discovering meaning. *TESOL Quarterly, 16*(2), 195–209.

**Appendix B (2000-2022 Corpus)**

Ávila-Cabrera, J. J., & Esteban, A. C. (2021). The project SubESPSKills: Subtitling tasks for students of Business English to improve written production skills. *English for Specific Purposes, 63*, 33-44.‏

Baker, B. A., Homayounzadeh, M., & Arias, A. (2020). Development of a test taker-oriented rubric: Exploring its usefulness for test preparation and writing development. Journal of Second Language Writing, 50, 100771.‏

Beck, S. W., Jones, K., Storm, S., & Smith, H. (2020). Scaffolding students’ writing processes through dialogic assessment. Journal of Adolescent & Adult Literacy, 63(6), 651-660.‏

Bowen, N. E. J. A., & Thomas, N. (2020). Manipulating texture and cohesion in academic writing: A keystroke logging study. *Journal of Second Language Writing, 50*, 100773.‏

Bychkovska, T. (2021). Effects of explicit instruction on noun phrase production in L2 undergraduate writing. *Journal of English for Academic Purposes*, 54, 101040.‏

Chien, S. C., & Li, W. Y. (2022). Problems of writing the doctoral dissertation discussion section: Advisors' and their doctoral students’ perspectives from natural and applied sciences and social sciences. *Journal of English for Academic Purposes, 60*, 101183.‏

Chuang, P. L., & Yan, X. (2022). An investigation of the relationship between argument structure and essay quality in assessed writing. *Journal of Second Language Writing, 56*, 100892.‏

Delgado-Osorio, X., Koval, V., Hartig, J., & Harsch, C. (2023). Strategic processing of source text in reading-into-writing tasks: A comparison between summary and argumentative tasks. *Journal of English for Academic Purposes, 62*, 101227.‏

Doolan, S. M. (2021). An exploratory analysis of source integration in post-secondary L1 and L2 source-based writing. *English for Specific Purposes, 62*, 128-141.‏

Du, H., & List, A. (2021). Evidence use in argument writing based on multiple texts. *Reading Research Quarterly, 56*(4), 715-735.‏

Fan, Y., & Xu, J. (2020). Exploring student engagement with peer feedback on L2 writing. *Journal of Second Language Writing, 50*, 100775.‏

Fang, Z., Gresser, V., Cao, P., & Zheng, J. (2021). Nominal complexities in school children’s informational writing. *Journal of English for Academic Purposes, 50*, 100958.‏

Fritz, E., Dormer, R., Sumi, S., & Kudo, T. (2022). The acquisition of formulaic sequences in EFL email writing. *English for Specific Purposes*, *65*, 15-29.‏

Graham, S. (2022). Creating a classroom vision for teaching writing. *The Reading Teacher, 75*(4), 475-484.‏

Granger, S., & Larsson, T. (2021). Is core vocabulary a friend or foe of academic writing? Single-word vs multi-word uses of THING. *Journal of English for Academic Purposes*, 52, 100999.‏

Jo, C. W. (2021). Short vs. extended adolescent academic writing: A cross-genre analysis of writing skills in written definitions and persuasive essays*. Journal of English for Academic Purposes, 53*, 101014.‏

Kim, H., & Rah, Y. (2021). Applying constructionist approaches to teaching English argument structure constructions to EFL learners. *TESOL Quarterly, 55*(2), 568-592.‏

Kocatepe, M. (2021). Reconceptualising the notion of finding information: How undergraduate students construct information as they read-to-write in an academic writing class. *Journal of English for Academic Purposes, 54*, 101042.‏

Labrador, B., & Ramón, N. (2020). Building a second-language writing aid for specific purposes: Promotional cheese descriptions. *English for Specific Purposes, 60*, 40-52.‏

Larsson, T., Reppen, R., & Dixon, T. (2022). A phraseological study of highlighting strategies in novice and expert writing*. Journal of English for Academic Purposes,* 60, 101179.‏

Lee, J. (2020). Effects of linguistic and affective variables on middle school students’ writing performance in the context of English as a foreign language: An approach using structural equation modeling. Reading and Writing, 33(5), 1235-1262.‏

Li, J., & Huang, J. (2022). The impact of essay organization and overall quality on the holistic scoring of EFL writing: Perspectives from classroom English teachers and national writing raters. *Assessing Writing, 51*, 100604.‏

Lin, L. H., & Morrison, B. (2021). Challenges in academic writing: Perspectives of engineering faculty and L2 postgraduate research students. *English for Specific Purposes, 63*, 59-70.‏

Liu, C., & Yu, S. (2022). Reconceptualizing the impact of feedback in second language writing: A multidimensional perspective. *Assessing Writing, 53*, 100630.‏

Maxwell-Reid, C., & Kartika-Ningsih, H. (2020). Nominal expansion in L2 adolescent writing: Functions and realizations of clausal embedding in argumentative texts. *Journal of Second Language Writing, 49*, 100751

Mochizuki, N. (2022). Multilingual doctoral students' lived experiences of genre knowledge development through social interactions: Learning writing ‘styles’ and ‘thought processes’. *Journal of English for Academic Purposes, 60*, 101181.‏

Mochizuki, N., & Starfield, S. (2021). Dialogic interactions and voice negotiations in thesis writing groups: An activity systems analysis of oral feedback exchanges. *Journal of English for Academic Purposes, 50*, 100956.‏

Omidian, T., Siyanova-Chanturia, A., & Biber, D. (2021). A new multidimensional model of writing for research publication: An analysis of disciplinarity, intra-textual variation, and L1 versus LX expert writing. *Journal of English for Academic Purposes, 53*, 101020.‏

Peltzer, K., Siekmann, L., Parr, J. M., & Busse, V. (2022). What beliefs about writing guide EFL curricula? An analysis of relevant policy documents for teaching English at German secondary schools. Zeitschrift für Erziehungswissenschaft, *25*(6), 1363-1387.‏

Rastgou, A. (2022). How feedback conditions broaden or constrain knowledge and perceptions about improvement in L2 writing: A 12-week exploratory study. *Assessing Writing, 53*, 100633.‏

Ryan, M., Khosronejad, M., Barton, G., Myhill, D., & Kervin, L. (2022). Reflexive writing dialogues: Elementary students’ perceptions and performances as writers during classroom experiences. *Assessing writing, 51*, 100592.‏

Saadatara, A., Kiany, G., & Talebzadeh, H. (2023). Bundles to beat the band in high-stakes tests: Pedagogical applications of an exploratory investigation of lexical bundles across band scores of the IELTS writing component. *Journal of English for Academic Purposes, 61*, 101208.‏

Sammour-Shehadeh, R., Kahn-Horwitz, J., & Prior, A. (2022). Spelling English as a foreign language: a narrative review of cross-language influences due to distance in writing system, orthography and phonology. *Reading and Writing, 36*(5), 1-27.‏

Shao, Z., Zhang, H., Zhang, J., Zhong, Y., & Xu, X. (2022). Phrasal complexity in English argumentative writing: Variations across Chinese STEM versus English majors' production and EFL textbook essays. Journal of English for Academic Purposes, 55, 101070.‏

Su, H., Zhang, Y., & Lu, X. (2021). Applying local grammars to the diachronic investigation of discourse acts in academic writing: The case of exemplification in Linguistics research articles. *English for Specific Purposes*, 63, 120-133.‏

Sun, Q., & Zhang, L. J. (2022). Examining the effects of English as a foreign language student-writers’ metacognitive experiences on their writing performance. Current Psychology, 42(9), 1-16.‏

Sun, T., Wang, C., & Wang, Y. (2022). The effectiveness of self-regulated strategy development on improving English writing: Evidence from the last decade. *Reading and Writing, 35*(10), 2497-2522.‏

Tabari, M. A. (2022). Investigating the interactions between L2 writing processes and products under different task planning time conditions. Journal of Second Language Writing, 55, 100871.‏

Teng, M. F., & Wang, C. (2021). Assessing academic writing self‐efficacy belief and writing performance in a foreign language context. *Foreign Language Annals.‏56(1)*, 144-169

Wang, M., & Zhang, Y. (2021). ‘According to…’: The impact of language background and writing expertise on textual priming patterns of multi-word sequences in academic writing. *English for Specific Purposes*, *61*, 47-59.‏

Wang, Y., & Xie, Q. (2022). Diagnosing EFL undergraduates’ discourse competence in academic writing. *Assessing Writing, 53*, 100641.‏

Westbrook, C. (2023). The impact of input format on written performance in a listening-into-writing assessment. Journal of English for Academic Purposes, 61, 101190.‏

Wingate, U., & Harper, R. (2021). Completing the first assignment: A case study of the writing processes of a successful and an unsuccessful student. *Journal of English for Academic Purposes*, *49*, 100948.‏

Xu, J., & Zhang, S. (2022). Understanding AWE feedback and English writing of learners with different proficiency levels in an EFL classroom: A sociocultural perspective. *The Asia-Pacific Education Researcher, 31*(4), 357-367.‏

Yang, C., Zhang, L. J., & Parr, J. M. (2020). The reactivity of think-alouds in writing research: Quantitative and qualitative evidence from writing in English as a foreign language. *Reading and Writing*, 33, 451-483.‏

Yang, M. (2023). Supervisory feedback, reflection, and academic discourse socialization: Insights from an L2 doctoral student's paper writing experience. *Journal of English for Academic Purposes, 62*,101215.‏

Yasuda, S. (2022). Natural scientists’ perceptions of authorial voice in scientific writing: The influence of disciplinary expertise on revoicing processes. *English for Specific Purposes, 67*, 31-45.‏

Yoon, H. J., & Tabari, M. A. (2023). Authorial voice in source-based and opinion-based argumentative writing: Patterns of voice across task types and proficiency levels. *Journal of English for Academic Purposes, 62*, 101228.‏

Zhang, M., & Plonsky, L. (2020). Collaborative writing in face-to-face settings: A substantive and methodological review. *Journal of Second Language Writing, 49*, 100753.‏

Zhu, X., Li, G. Y., Cheong, C. M., Yu, G., & Liao, X. (2021). Secondary school students’ discourse synthesis performance on Chinese (L1) and English (L2) integrated writing assessments*. Reading and Writing, 34*, 49-78.‏

**Appendix C: Sample 1**

The Composition/Conversation Class Is Too Heterogeneous Another problem that renders the teaching of writing difficult, if not impossible, is the heterogeneity of the composition/conversation

class. Because of diverse reasons for taking an intermediate or advanced composition/conversation course (e.g., requirements, field of specialization, electives), the heterogeneous nature of

the class is more salient at the college level. Students are widely different in their perceptions,

experiences, intellectual and academic levels, and all influence the scope, the depth, and the efficiency of the verbal process. A study conducted by V. Book revealed that written messages produced by students characterized as high or low apprehension differed significantly in structure,

language use, and amount of information conveyed.) Other studies have shown that extroverts

may be too impulsive in their written expression to distinguish between relevant and irrelevant details and may therefore pack their composition with irrelevant information. Surely students who have never travelled outside their native state may encounter difficulties when asked to describe the sentiments and the excitement that accompany a trip overseas. Clearly, the objectives of a student taking an intermediate or advanced composition/conversation course to meet language requirements will be different from those of a student whose goal is to become a French teacher.

Although there exists no magic method to produce "good" writers, the above examples suffice

to convince us of the need to improve writing instruction in second language programs. Ideas and

techniques promoted by departments of English may be of interest to second language teachers.

Developing The Writer

If we are willing to admit that the purpose of writing is communication, it follows that the inclusion of a well-designed writing program is necessary in the second language curriculum. Such a program must be designed to guide the student from basic writing skills to advanced composing. In other words, there must be a gradual building up from material reduced initially to a series of frames (e.g., sentence-builders, dehydrated sentences, open-ended sentences) to fully developed concepts such as the complete sentence, the well-constructed paragraph, and finally, the whole composition. Its ultimate aim must be the development of mature writers; that is, writers who are able to clearly communicate a message. In second language programs, however, instruction stops at the complete sentence and never reaches the stages of the composing process rewriting, writing, reconsidering, editing). The importance of developing the stages of the com- posing process has been emphasized by B.Μ. Kroll. Kroll sees the first two (prewriting and writing) as the reader's role. The mature writer is one who can assume both roles with confidence.

While the writer's role may appear simple and familiar, it is a complex and serious task that goes beyond the grammatical and syntactic technicalities of the sentence. It is a part of the composing process where the writer identifies ideas and endeavors to express them in the written language. As Kroll states, it is "an initial period of finding something to say (prewriting), followed by a formal writing-down of ideas in a draft (writing)."s This initial stage is of importance to the second language writer. Unless guided and encouraged in prewriting, students launch into ideas they cannot express either because of their linguistic limitations or their inability to retrieve the second language fast enough.

Although there are many techniques for developing the writer’s role, P. Elbow’s freewriting exercises can be used most effectively in the second language classroom.

**Appendix D: sample 2**

In this exchange, we saw Nanika’s reasoning around “an easy enough word” as a strategic rejection of more conventionally academic suggestions in favor of linguistic accessibility. Elsewhere in the session, she had skillfully deployed many literary terms, such as iambic pentameter and rhyme scheme, and argued that such technical vocabulary was necessary for her audience to understand her literary interpretation. However, when referring to human characteristics, she opted for the more colloquial word feelings. Notably, Scott did not neutrally accept Nanika’s choice but elicited her ideas about why this was a better choice, and after she provided a well-reasoned critique, he provided a strong endorsement (“go for it!”). In this moment, Scott’s mediational moves did not neatly align with the established epistemologies but, instead, suggested an epistemology concerned with teaching students how to both use and resist disciplinary conventions in a principled way.

Discussion

In this study, we explored how three teachers varied their scaffolding of students’ composing processes in response to students’ individual needs, and how their scaffolding—through mediational moves and written tools—reflected both explicit, conscious goals and implicit epistemologies. Importantly, our study suggests that the mediational moves are not aligned with specific epistemologies; rather, any mediational move can be leveraged as a verbal scaffold to support varying goals. For example, Ms. Miller used the move of checks understanding to support structural work, whereas Holly used it to support an ideational focus. These moves provide a set of tools that teachers can use as verbal scaffolds in dialogic assessment conferences. The work of these teachers supports a view of scaffolding that is dynamic and relational (Smagorinsky, 2018b) rather than static and standardized. Their work also suggests that the framework of epistemologies for writing instruction (Newell et al., 2014) would benefit from expansion and elaboration to better represent the complexity and comprehensiveness of all that is involved in teaching writing. In particular, the teachers’ work provides evidence to support expanding the epistemologies framework in two ways: by including an affective-relational dimension to the social practice epistemology and by adding a fourth epistemology related to transformative critique of disciplinary norms for academic discourse, as evidenced in Scott’s teaching. Although it was not our intention to distinguish between better and worse ways of scaffolding students’ writing processes through dialogic assessment, we noticed some differences among the teachers that corresponded to differences in their years of experience. Both Scott and Holly, the more experienced of the three, foregrounded different epistemologies depending on students’ needs. Scott chose scaffolds reflecting a structural epistemology with Gaspar but not with the others, because Gaspar had many ideas but needed help in organizing them to make deeper claims. Holly, although she frequently seemed to draw on a relational epistemology, invoked a structural epistemology through metalinguistic clues when working with Julia, because she was attuned to this student’s need for extra linguistic support as a former emergent bilingual. In contrast, Ms. Miller’s scaffolding practices more consistently reflected a structural epistemology. The teachers’ different ways of scaffolding also seemed to imply different conceptions of progress. For example, Ms. Miller’s instruction focused on students’ adherence to a set of structural organizers aligned with their local classroom rubric, whereas Holly’s relational work with students was informed by future collegiate contexts for writing.
